# Hyperthyroidism and Jaundice

**DOI:** 10.4103/0972-3919.78244

**Published:** 2010

**Authors:** CS Bal, Madhavi Chawla

**Affiliations:** Department of Nuclear Medicine, All India Institute of Medical Sciences, New Delhi, India

**Keywords:** Hyperthyroidism, jaundice, anti-thyroid drugs, anti-tubercular drugs

## Abstract

Development of hyperbilirubinemia, concurrent or subsequent to hyperthyroidism, can be due to thyrotoxicosis *per se*, or due to drug treatment of hyperthyroidism. Other rare conditions: autoimmune thyroid disease, or causes unrelated to hyperthyroidism like viral hepatitis, alcohol abuse, sepsis, cholangitis, or as a side effect of certain medications. In this article, we review these causes of co-existent hyperthyroidism and jaundice. We also highlight the changes to be expected while interpreting thyroid function tests vis-a-vis liver function tests in this subgroup of patients.

## INTRODUCTION

Hepatic dysfunction was described in patients with hyperthyroidism for the first time in 1874.[1] The association between the liver and thyroid diseases has been a subject of intense investigation over a century; however, the exact nature of the relationship remains elusive. The cause of hepatic dysfunction in hyperthyroidism may be multifactorial, occurring as a result of hyperthyroidism per se, drug treatment of hyperthyroidism, conditions associated with autoimmune thyroid disease, and, rarely, alteration of thyroid hormone metabolism secondary to intrinsic liver disease such as Gilbert’s syndrome. It can also result from causes unrelated to hyperthyroidism such as viral hepatitis, alcohol abuse, sepsis, cholangitis and medications such as oral contraceptives, acetaminophen, isoniazid and rifampicin.[2] The symptoms can range from mild liver dysfunction and gastrointestinal symptoms to serious liver dysfunction like fulminant hepatitis.[3] Here, we review the various causes of coexistent hyperthyroidism and hepatic dysfunction, with emphasis on jaundice due to antithyroid drugs.

## JAUNDICE DUE TO HYPERTHYROIDISM *PER SE*

Hyperthyroidism per se manifesting as clinical icterus is extremely rare, if at all patients have minimal jaundice. There are several hypotheses regarding the cause of cholestasis in thyrotoxicosis. The hypermetabolic state in hyperthyroidism increases the hepatic oxygen consumption without increasing the hepatic blood flow, thus lowering the oxygen tension in the centrilobular zones and interfering with bile transport, probably resulting in cholestasis and increasing the rate of bile flow to the point of saturation.[4–6] It is suspected that excess thyroxine can produce direct toxic effect on hepatocytes; however, studies have not confirmed this.[5]

## JAUNDICE DUE TO ANTITHYROID DRUGS

Methimazole (MMZ), its prodrug carbimazole (CBZ), and propylthiouracil (PTU) have been used over several decades for the control of hyperthyroidism. MMZ is recommended as the first line drug for the treatment of hyperthyroid therapy, an exception to this being pregnancy, where PTU is used because of the higher incidence of birth defects associated with MMZ. Another scenario where PTU is preferred is in patients with severe, life-threatening thyrotoxicosis because of its additional inhibition of T4 to T3 conversion. PTU, however, needs to be given with caution due to the increasing reports of PTU related liver failure and death, especially in children.[7] The average daily dose of PTU associated with liver failure is approximately 300 mg in both adults and children. The reported hepatic injuries present as jaundice and are usually histologically severe and are sometimes associated with hepatic failure and death.[8,9] Discontinuation of the drug leads to complete recovery in most cases.[10,11] Abnormalities or worsening of liver function test (LFT) suggest the diagnosis. Elevations in liver enzymes are seen in about 30% of patients after 2 months of therapy, with no elevation of the bilirubin level (transaminitis)[12] and despite continuation of therapy, has been seen to normalise in most patients by 5 months.[13] Pathological findings range from early signs of hepatocellular inflammation and swelling to submassive hepatic necrosis. Nonspecific hepatocellular necrosis is typically found on liver biopsy.[8,11]

MMZ, on the other hand, has only been documented to cause liver function abnormality in a handful of cases.[14] MMZ-induced jaundice appears to be a hypersensitivity reaction occurring in patients receiving normal doses of the drug. Jaundice due to MMZ is usually associated with cholestatic liver function tests and histological features on liver biopsy of cholestasis and non-specific infiltration of portal tracts with mono-nuclear cells.[14] Hypersensitivity is suggested by the occasional finding on liver biopsy of eosinophils infiltrating the portal tracts. An elevation of bilirubin is the major abnormality. Mild elevations in liver enzymes and bilirubin occurs within 2 weeks of initiation of MMZ therapy and are associated with cholestasis on liver biopsy, with minimal, if any, cellular damage.[14]

## JAUNDICE IN THYROCARDIACS

Atrial fibrillation and thyrotoxic heart failure are two clinically important effects of thyrotoxicosis. Thyrotoxicosis is known to aggravate pre-existing heart disease and also lead to heart failure in a person with unknown existing heart disease.[15] Jaundice, in the past, had been thought to be related to hyperthyroidism complicated by congestive heart failure and secondary hepatic dysfunction. Although the pumping action and filling capacity of the heart may be normal, abnormally high oxygen demand by the body tissues make it difficult for the heart to supply an adequate blood flow, resulting in high-output heart failure. Fluid accumulates in the liver, thereby impairing its ability to rid the body of toxins and produce essential proteins. The increase in bilirubin level is generally due to increased level of conjugated bilirubin. Atrial fibrillation occurs in 10-15% of elderly patients with long-standing uncontrolled hyperthyroidism[16] and is associated with significant morbidity resulting from embolic events[17] rather than hepatic failure.

## JAUNDICE IN ASSOCIATION WITH OTHER AUTOIMMUNE DISEASES

Autoimmune hepatitis (AIH) and primary biliary cirrhosis (PBC) are autoimmune diseases, which may coexist with thyrotoxicosis. AIH is a condition charecterised by anomalous presentation of human leucocyte antigen class II on the surface of hepatocytes, resulting in a cell-mediated immune response against the body’s own liver. Particularly type 2 AIH is associated with a wide variety of other immunologic disorders including thyrotoxicosis. Involvement of other systems may present at disease onset or during the course of active liver disease. As with most other autoimmune diseases, it affects women more often than men. AIH should be suspected in any young patient with hepatitis, especially those without risk factors like alcoholic, drug, metabolic, viral or hereditary etiologies. The workup of AIH should include testing for serum antinuclear antibodies (ANA), anti-smooth muscle antibodies (ASMA), serum protein electrophoresis, and quantitative immunoglobulin. Serum protein electrophoresis and testing for autoantibodies are of central importance in the diagnosis of AIH. Patients in whom a diagnosis of AIH is suspected should have a liver biopsy done.

Primary biliary cirrhosis is a chronic cholestatic liver disease of autoimmune origin, characterised by inflammatory destruction of the small bile ducts within the hepatic parenchyma, eventually leading to cirrhosis. Like thyroid disorders, most patients with PBC are middle-aged women.[18,19] More than half of patients are asymptomatic at diagnosis.[20,21] They are generally identified by finding of a marked elevation of serum alkaline phosphatase (SAP) and gamma glutamyltranspeptidase (GGTP), the enzymes present in the bile ducts and moderate elevation of serum alanine aminotransferase and aspartate aminotransferase. An elevation in serum bilirubin levels and total gamma-globulin levels is seen as the disease progresses. In addition, elevated levels of serum cholesterol and serum IgM concentration are present. Serum autoantibodies are of primary importance in the diagnosis of PBC. The serological hallmark of the disease is the presence of circulating antimitochondrial antibodies (AMA), which can be detected in nearly 100 % of patients, by using sensitive diagnostic methodologies based on recombinant antigens.[22] High titres of disease-specific ANAs are also seen. Diagnosis can be typically established by the triad of AMA, cholestatic indices, and liver histology diagnostic or compatible with PBC. Histologically, infiltration of presumably autoreactive T cells in the liver and periductular spaces is one of the major features of the disease.[23,24]

## SIMPLE ASSOCIATION OF INHERITED HYPERBILIRUBINAEMIAS AND HYPERTHYROIDISM

People with Gilbert’s syndrome have mild, chronic unconjugated hyperbilirubinaemia in the absence of liver disease or overt haemolysis, and is found in up to 2-13% of the population.[25,26] The major symptom is otherwise harmless jaundice, which does not require treatment. The source of this hyperbilirubinaemia is reduced activity of the enzyme glucuronyltransferase, which conjugates bilirubin. Conjugation renders the bilirubin water-soluble, after which it is excreted in bile into the duodenum. Gilbert’s syndrome is caused by approximately 30-50% reduced glucuronidation activity of the enzyme Uridine-diphosphate-glucuronosyl transferase isoform 1A1 (UGT1A1). Mild jaundice may appear under conditions of exertion, stress, fasting, and infections, but the condition is otherwise asymptomatic. Laboratory investigations reveal hyperbilirubinaemia, the bilirubin levels in the range of 1.2-5.3 mg/dL, with predominantly unconjugated hyperbilirubinaemia and a ratio of unconjugated/conjugated bilirubin that is commensurately higher than normal.[25] The level of total bilirubin is often exacerbated upon fasting.[27] The transaminases are usually within the normal range. The serum bilirubin levels rise during the hyperthyroid phase and return to baseline level upon treatment with antithyroid drugs. Thus, Gilbert’s syndrome per se should not be a contraindication for radioiodine therapy for hyperthyroidism. Hence, interactions between thyroid and hepatic disease as well as the patient’s clinical status must be considered in the interpretation of thyroid function tests and the management of hyperthyroidism in these settings.

## ABOUT THYROID FUNCTION TESTS

Special care must be taken for the measurement of thyroid function tests (TFT) in co-existent thyrotoxicosis and jaundice. Total T3 and T4 levels alone may not reflect the patient’s true thyroid status. Acute hepatitis increases the concentration of serum thyroid hormone-binding globulin (TBG), causing an increase in the total T_4_ level and a decrease in the thyroid hormone binding ratio.[28] With progressive hepatic dysfunction, the interaction between thyroid and hepatic disease becomes even more important. An inverse linear relationship has been observed between total T_4_ level and serum bilirubin.[13,29] Low total T_4_ and markedly elevated free T4 levels may be seen due to a decreased concentration of thyroid hormone-binding proteins,[30,31] suggesting persistent and insufficiently treated hyperthyroidism. Bilirubin can also interfere with the measurement of T_4_ by lowering the affinity of T_4_ for thyroid hormone-binding proteins.[32] Also, free T_4_ may be elevated when total T_4_ is normal or low in hyperthyroidism associated with severe illness.[33] The measurement of free T_4_ levels may aid in the interpretation of the patient’s thyroidal status.

## LIVER FUNCTION TESTS IN CO-EXISTENT THYROTOXICOSIS AND JAUNDICE

Up to 72% of patients with hyperthyroidism and presumably normal liver function may have an elevation of at least one hepatic enzyme.[34] Serum alkaline phosphatase (SAP) elevations are most commonly reported,[35,36] with predominant elevation of bone isoenzyme, increased osteoblastic activity resulting from hyperthyroidism being responsible for the elevation of SAP in most cases.[35,37] Transaminase elevations may be due to thyrotoxicosis-induced increased hepatic oxygen consumption,[4,38] with inadequate compensatory hepatic blood flow.[4] Elevation of GGTP levels are seen in 16.8-62% patients in various studies,[34,39] and usually correlates well with SAP.[39] Besides the above conditions, certain unrelated conditions and medications can also cause associated jaundice in patients with hyperthyroidism.
